# Electrode impedance dynamics in sequential cochlear implant users: insights into cochlear immunity

**DOI:** 10.3389/fimmu.2025.1702549

**Published:** 2025-10-29

**Authors:** Logan L. Flom, Eva L. Rasche, Jacob J. Oleson, Rachel A. Scheperle, Marlan R. Hansen

**Affiliations:** ^1^ Department of Otolaryngology-Head and Neck Surgery, University of Iowa Health Care Medical Center, Iowa City, IA, United States; ^2^ Department of Biostatistics, University of Iowa, Iowa City, IA, United States

**Keywords:** cochlear implant, impedance, inflammation, immunology, hearing loss prevention, foreign body response, immunological memory, retrospective cohort

## Abstract

**Introduction:**

Cochlear implant outcomes can be limited due to immunologically mediated intracochlear foreign body responses, resulting in new bone growth and fibrosis. Minimal consideration has been given to the possible role of immunological memory in modulating this response in sequentially implanted patients. We hypothesize the first implant primes the contralateral ear to respond more robustly to sequential implantation, leading to earlier increases in electrode impedance.

**Methods:**

This is a retrospective cohort analysis of clinical impedance measurements from 79 subjects with sequential bilateral implants. Raw impedance and changes in impedance were analyzed over time according to implant sequence.

**Results:**

Paired *t*-tests comparing 12-month average absolute impedance between implants were statistically significant (22 electrodes, *p* = 0.0176; 95% confidence interval [CI] = − 731.37, − 71.84; excluding five basal electrodes, *p* = 0.0070; 95% CI = − 784.31, − 128.40). Linear mixed models showed significant effects at *p* < 0.0001, including implant sequence, time elapsed, and electrode grouping. Estimated marginal means revealed statistically significant differences in delta impedance between all combinations of basal, middle, and apical subsets. Within each subset, statistically significant differences in delta impedance by implant sequence were observed in the basal (*p* = 0.0136) and apical (*p* = 0.0067) groups. Estimated marginal slopes of delta impedance by implant sequence were also significantly different (*p* < 0.0001).

**Discussion:**

More rapid increases and greater electrode impedances are consistent with a more robust immune response in the second implanted ear. Additional investigation into the effects of implant timing, electrode array type, perioperative corticosteroids, and complex impedances may further elucidate these relationships and their implications for the cochlear immune response.

## Introduction

Hearing loss represents one of the primary sources of both physical and financial morbidity in the modern era of healthcare. It affects a quarter of adults over the age of 45 and nearly 800,000 infants each year. Hearing loss has been estimated to cost the global economy more than $750 billion annually due to lost productivity, healthcare costs, and educational support systems, with up to $194 billion of these costs arising from the USA alone (1, 2). When left untreated, the consequences are deleterious and include worse outcomes in speech and language development, cognitive abilities, and quality of life in both infants and adults (3, 4). Presbycusis, characterized by age-related progressive bilateral symmetrical sensorineural hearing loss, increases in prevalence each decade, from 5%–10% at age 40 to 80%–90% at age 85, reinforcing the importance of early intervention (4, 5).

One option for individuals with severe-to-profound hearing loss is cochlear implantation, which consists of a surgically implanted intracochlear electrode array that directly stimulates the auditory nerve to elicit auditory perception (6). These neuroprosthetic devices provide significant improvements in auditory function, detection, speech perception (in quiet and noise), sound localization, and quality of life (7–10). However, cochlear implant outcomes vary, with some recipients performing worse than expected due to multiple factors, including insertion trauma, intracochlear tissue responses (fibrosis, neo-ossification), and inflammation (7). While the materials used for cochlear implants are biocompatible, they are not bioinert and consistently elicit an inflammatory foreign body response that can constrain hearing outcomes (11, 12).

Foreign body responses are based on the underlying immunology. Similar to the identification of pathogen-associated molecular patterns (PAMPs) in the recognition of microbial pathogens, the immune system also responds to signals arising from inflammation, ischemia, and hypoxia, known as damage-associated molecular patterns (DAMPs) (13). These pathways are closely related to the concept of sterile inflammation, activating the innate and adaptive immune responses (14). Innate immune cells, including macrophages, dendritic cells, neutrophils, and mast cells (among others), release inflammatory mediators and directly participate in the clearance of affected cells through phagocytosis and degranulation, which activates adaptive T cells and B cells, promoting cytotoxicity and antibody production (15, 16). Therefore, the placement of a foreign body (such as a cochlear implant) often leads to sterile inflammation and results in an immune signaling cascade aimed at mounting an effective response. In cochlear implants, this intracochlear tissue response manifests as fibrosis and new bone growth within the scala tympani surrounding the electrode array.

However, adaptive immunological memory, characterized by the immune system’s ability to remember encountered PAMPs or DAMPs and subsequently respond faster and more robustly than before (17), is relatively unexplored in cochlear environments. While immunological memory is traditionally viewed in the context of adaptive memory T and B cells, five distinct types of immunological memory, including some within the innate arm, have been identified. Special cases of immunological memory in the context of autoimmunity development include sympathetic ophthalmia, in which damage to one eye effectively “spreads” to the other eye despite underlying immune privilege that typically separates these sites (18). Though the inner ear was historically thought to demonstrate similar immune privilege as the eye, studies have revealed robust inflammatory and immune responses within the cochlea (19).

While several studies have characterized the foreign body response resulting from cochlear implantation (20–27), minimal consideration has been given to the potential role of immunological memory in mitigating or exacerbating this response, potentially contributing to contralateral priming in sequentially implanted cochlear implant users. Currently, there are no clinical measures to quantify cochlear inflammation and fibrosis following electrode array placement. Electrode impedance changes have been shown in animal models to track with these tissue changes and are frequently used as a marker for intracochlear tissue remodeling after cochlear implantation (28–30). Using electrode impedance as a sensitive measure of inflammation, fibrosis, or new bone formation (11, 28, 31–39), we hypothesize that the first implant primes the contralateral ear to respond more robustly to sequential implantation, leading to earlier increases in electrode impedance than the first implant.

## Materials and methods

### Study design and approval

This study is a retrospective cohort analysis of clinical impedance measurements from the University of Iowa Health Care Cochlear Implant Center. A total of 2,363 unique patient profiles, with associated demographic and electrophysiology data, were identified within the University of Iowa Cochlear Custom Sound SQL database and extracted for analysis on 31 July 2024. All research procedures adhered to ethical guidelines for human subject research and received approval from the Institutional Review Board (IRB No. 202404918) prior to investigation.

### Inclusion and exclusion criteria

**Figure 1:**
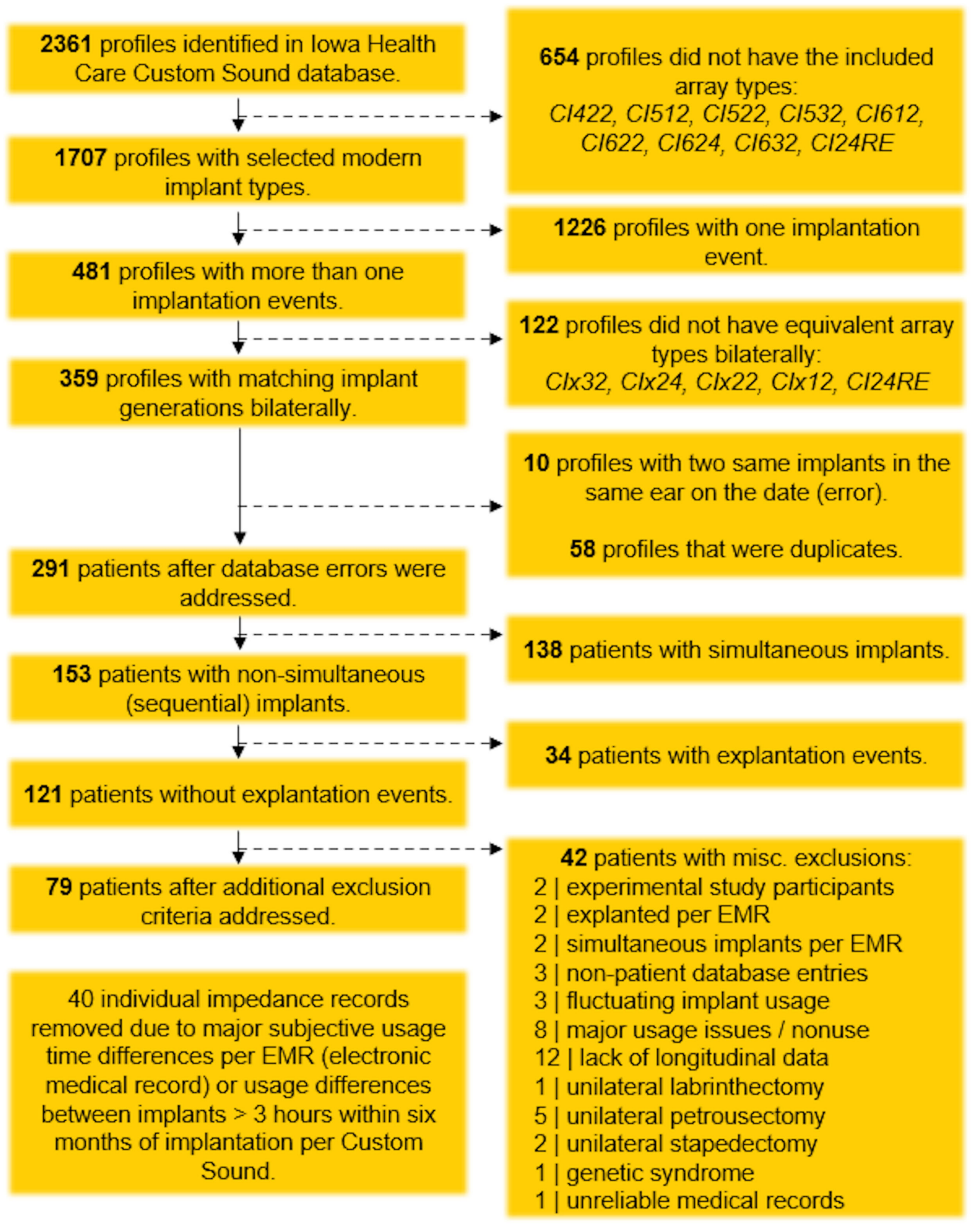
Flowchart of data-cleaning process, illustrating the application of inclusion and exclusion criteria.

To maximize the potential elucidation of our hypothesis, strict inclusion and exclusion criteria were applied using sequential MATLAB (R2023b–R2024b) scripts, with the processing steps described in **Figure 1**. Inclusion criteria included patients with bilateral cochlear implants that were sequentially implanted on separate dates rather than simultaneously, Cochlear CI24RE or newer implants, similar array types across ears, and no explantation events. After serial processing, 121 unique patient profiles remained. Further review of all pertinent medical and audiological records using the electronic medical record revealed additional factors that could confound impedance outcomes, leading to additional exclusion criteria (also detailed in **Figure 1**) and a final cohort of 79 unique patients for data analysis.

### Data visualization and analysis

The final dataset included patient and implant identifiers; dates associated with implantation, initial stimulation, and impedance recordings; and impedance measurements and deactivation status across all 22 electrodes. The MP2 stimulation mode was selected for impedance measurements because of the stability of the case ground and its relatively consistent effects on each intracochlear electrode. For each deactivated electrode, impedance measurements were removed from the visit of initial deactivation through the end of the patient’s record. This decision was intended to minimize potentially confounding effects of electrode position (for example, when the most basal electrode was extracochlear) and current-carrying status. An additional 40 individual impedance records were removed due to major subjective differences in usage time, based on audiologic visit records, or differences between implants greater than 3 hours within 6 months of implantation, according to Custom Sound records.

As impedance transiently increases from surgery to initial activation and subsequently decreases with electrical stimulation, impedance data were analyzed relative to the baseline visit, defined as the visit immediately following initial activation, typically 2 weeks (29, 40–42). Summary statistics were calculated for age at implant, time since baseline, time between implants, impedance at the baseline visit, absolute impedance relative to baseline visit, and delta impedance relative to baseline visit. For each patient, absolute impedance was averaged across all 22 electrodes and all 22 electrodes minus the five basal electrodes, and these averages were plotted against time. This latter model was included because audiologists may deactivate basal electrodes for reasons (e.g., aversive sound quality) unrelated to our hypothesis. Average absolute impedance in the first 12 months from the baseline visit, by implant sequence, for all 22 electrodes and for all 22 electrodes minus the five basal electrodes, was visualized using violin plots. The mean change was analyzed using paired *t*-tests at a 95% significance level.

Electrodes were grouped into three anatomically based subsets: basal (3–6), middle (10–13), and apical (19–22). Subsets were selected by choosing four sequential electrodes at the ends and in the middle of the electrode array to better delineate regional and anatomical differences without overcomplicating subsequent models (43). Using the entire dataset, delta impedance was plotted against time from baseline for each group. A linear mixed model was then used to characterize relationships between delta impedance (in Ohms) and the following variables. Fixed effects included electrode grouping (basal, middle, apical), implant sequencing (first implant, second implant), age (binary variable to age < 18 or ≥ 18), and time since baseline (in months). The time variable was modeled on the log scale to capture nonlinear trajectories, as impedance changed rapidly after implantation and then stabilized. Interactions were included between group and implant sequence, implant sequence and log-transformed time, and group by log-transformed time. Random effects included a random intercept for each participant and a random slope for log-transformed time, allowing individuals to have their own trajectories and accounting for the correlation from examining both ears. Analyses were performed in R v4.4.2 using the *nlme* package for modeling and the *emmeans* package for specific comparisons of interest (44–46).

Individual effects were evaluated using *t*-tests, while pairwise comparisons within each electrode group were performed using *t*-tests on estimated marginal means. Contrasts were calculated to compare the implant sequence for each electrode grouping. Estimated marginal slopes for each implant sequence (averaged over the levels of electrode group and age category) were compared using *t*-tests on the estimated marginal means of delta impedance over time by implant sequence. Estimated marginal slopes for each electrode grouping (averaged over implant sequence and age category) were compared using *t*-tests on the estimated marginal means of delta impedance over time by electrode group. A Tukey multiple comparisons adjustment was applied to each set of pairwise comparisons.

## Results

### Patient demographics and summary statistics

General summary statistics for patient demographic information, including sex, age at implant, age group, implant types, time between implants, time since baseline visit, impedance at baseline visit, absolute impedance relative to baseline visit, and delta impedance relative to baseline visit, are presented in **Tables 1**, **2**.

**Table 1:** Description of patient demographic data with summary statistics, including sex, age at implant (in years), age group, implant types, time between implants (in years), and time since baseline visit (in years).

Variable	*N*	Mean	SD	Min	Q1	Q3	Max
Sex	79						
Male	44						
Female	35						
Age grouping	79						
< 18 years	42						
> 18 years	39						
Age at implantation	158	27.60	28.69	0.85	2.63	11.27	80.14
Implant 1	79	27.00	28.52	0.85	2.24	11.17	77.67
Implant 2	79	28.20	29.03	1.03	2.74	60.31	80.14
Time between implants	158	1.20	1.22	0.13	0.44	1.48	6.23
Time since baseline visit	1,686	2.56	3.31	0	0.28	3.64	18.30
Implant 1	983	2.54	3.23	0	0.36	3.50	18.30
Implant 2	703	2.59	3.43	0	0.22	3.68	16.30
**Implants**	**CI422**	**CI512**	**CI522**	**CI532**	**CI624**	**CI632**	**CI24RE (CA)**
Implant 1	10	11	3	20	2	27	6
Implant 2	10	11	3	12	2	35	6

*N* (for sex and age grouping), number of subjects; *N* (for age at implantation, time between implants, and implant types), number of implants; *N* (for time since baseline visit), number of visits; SD, standard deviation.

Bolded implant subheadings denote distribution of implant types.

**Table 2:** Description of patient impedance data with summary statistics, including electrode impedance at baseline visit, absolute impedance relative to baseline visit, and delta impedance relative to baseline visit (in Ohms).

Variable	*N*	Mean	SD	Min	Q1	Q3	Max
Baseline impedance	35,996	9,156	2,166	3,847	7,729	10,186	18,466
Implant 1	20,997	9,069	2,251	3,894	7,590	10,088	18,466
Implant 2	14,999	9,277	2,035	3,847	7,990	10,284	17,652
Absolute impedance	35,996	8,983	2,668	2,367	7,130	10,408	26,937
Implant 1	20,997	8,857	2,760	2,367	6,937	10,337	26,937
Implant 2	14,999	9,159	2,523	3,160	7,458	10,514	23,173
Delta impedance	35,996	− 172.79	2,671	− 11,246	− 1,558	1,024	15,323
Implant 1	20,997	− 212.06	2,738	− 11,246	− 1,591	1,049	15,323
Implant 2	14,999	− 117.81	2,575	− 8,613	− 1,516	983.50	14,272

*N*, number of nondeactivated electrodes; SD, standard deviation.

### Absolute impedance outcomes

**Figure 2:**
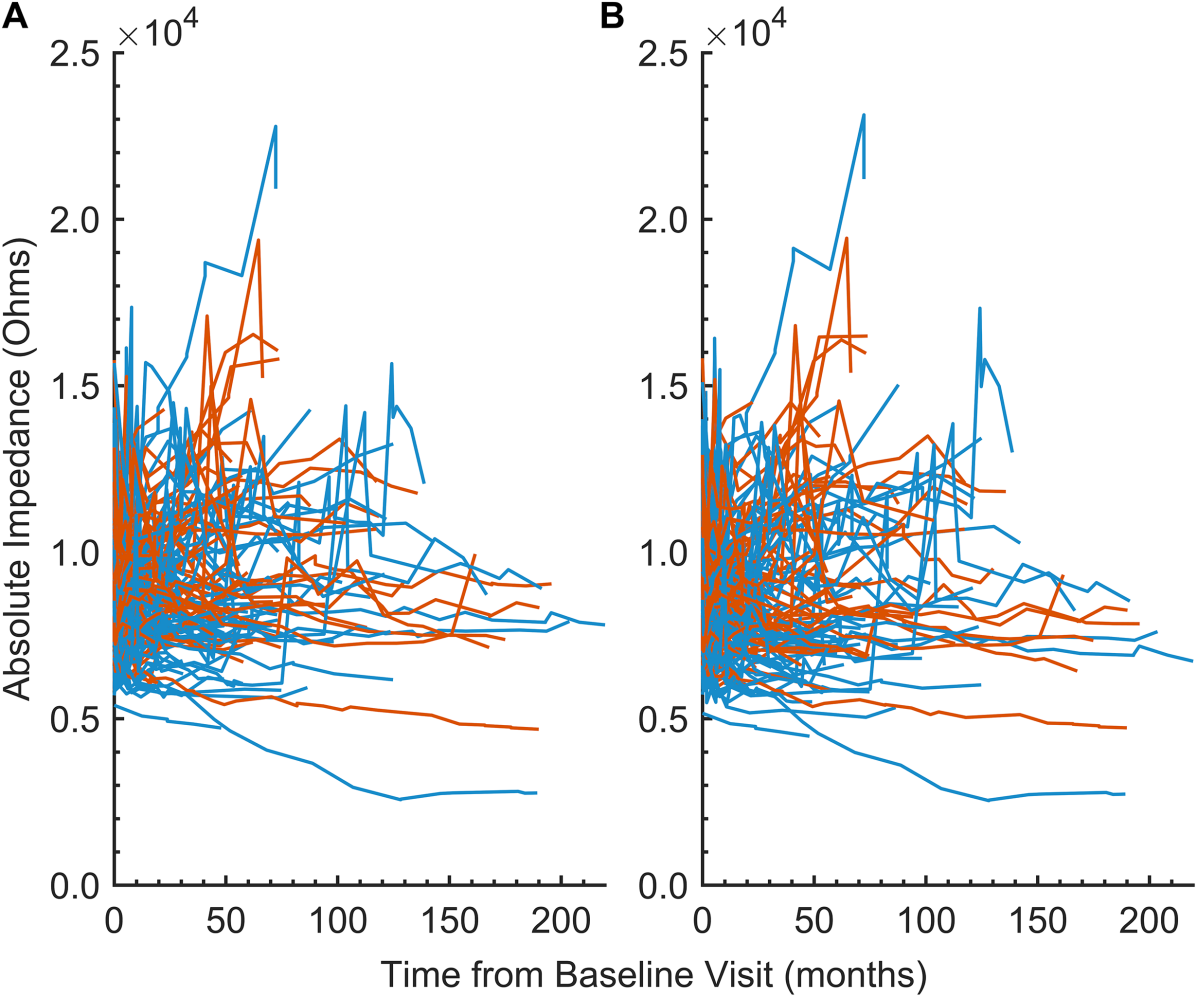
**(A)** Line plot of the 12-month average absolute impedance of all 22 electrodes (in Ohms) versus time since baseline visit (in months) by implant 1 (blue) and implant 2 (orange), demonstrating within- and between-subject variability. Each line represents an individual subject in the cohort. **(B)** Similar to **(A)**, but excluding electrodes 1–5 from the average.

**Figure 3:**
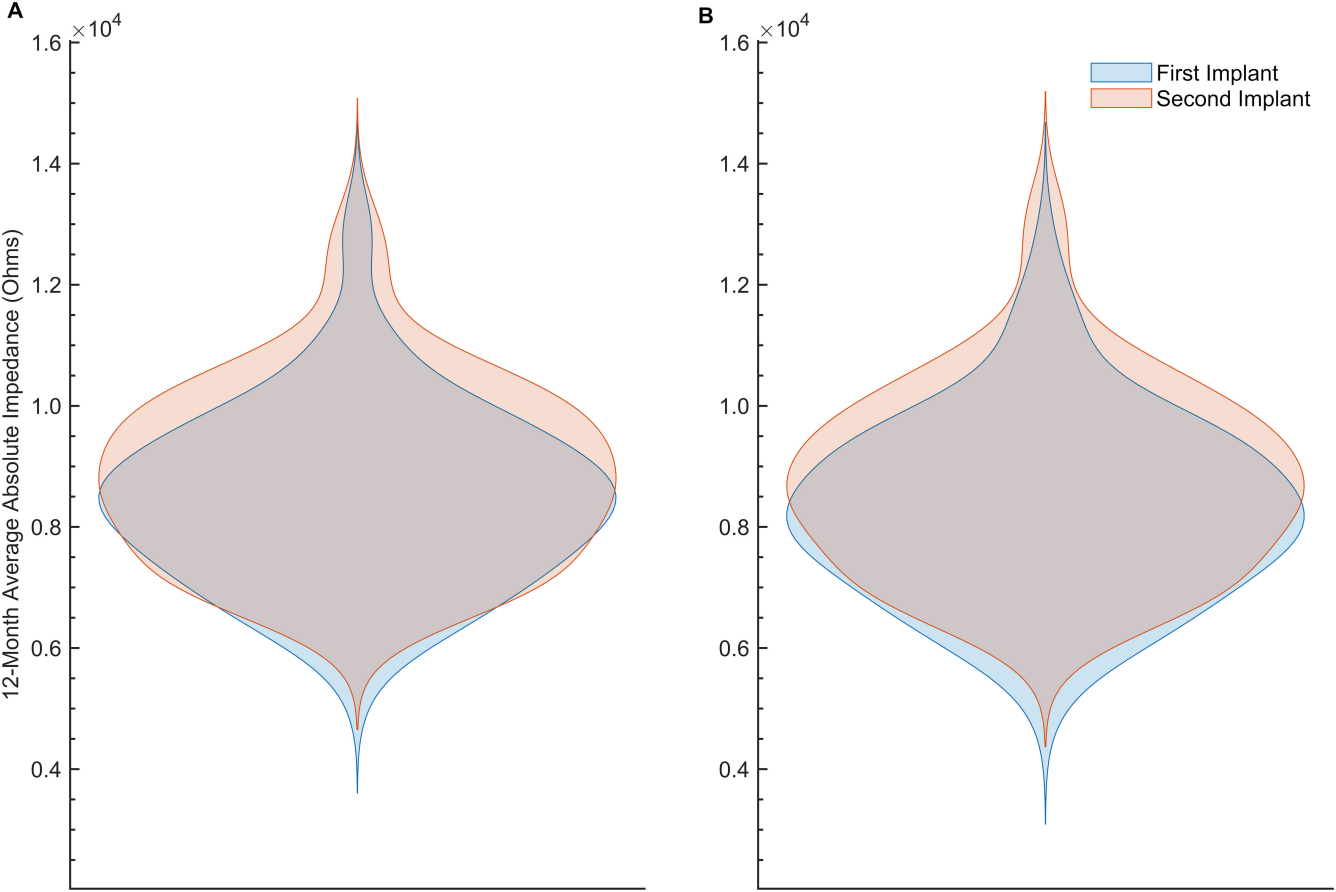
**(A)** Violin plot of the 12-month average absolute impedance of all 22 electrodes (in Ohms) versus time since baseline visit (in months) by implant 1 (blue) and implant 2 (orange). **(B)** Similar to **(A)**, but after excluding electrodes 1–5 from the average.

Given that tissue responses to cochlear implantation display both temporal and spatial dynamics (47), we examined impedance changes over time and across anatomically discrete regions of the cochlea. The average absolute impedance for all 22 electrodes (**Figure 2a**) and for all 22 electrodes minus the five basal electrodes (**Figure 2b**) was plotted against time since baseline visit for the entire dataset. These distributions were then focused on the first 12 months postimplantation and visualized using violin plots (**Figure 3**). Paired *t*-tests comparing the 12-month average absolute impedance for all 22 electrodes between the first implant and second implants were statistically significant (*t* = − 2.42, degree of freedom [*df*] = 78, *p* = 0.0176; 95% confidence interval [CI] = − 731.37, − 71.84). Paired *t*-tests comparing the 12-month average absolute impedance for all 22 electrodes minus the five basal electrodes between first and second implants were also statistically significant (*t* = − 2.77, *df* = 78, *p* = 0.0070; 95% CI = − 784.31, − 128.40). The corresponding confidence intervals for both models indicate higher 12-month average absolute impedances in the second implant.

### Delta impedance outcomes and statistical modeling

**Figure 4:**
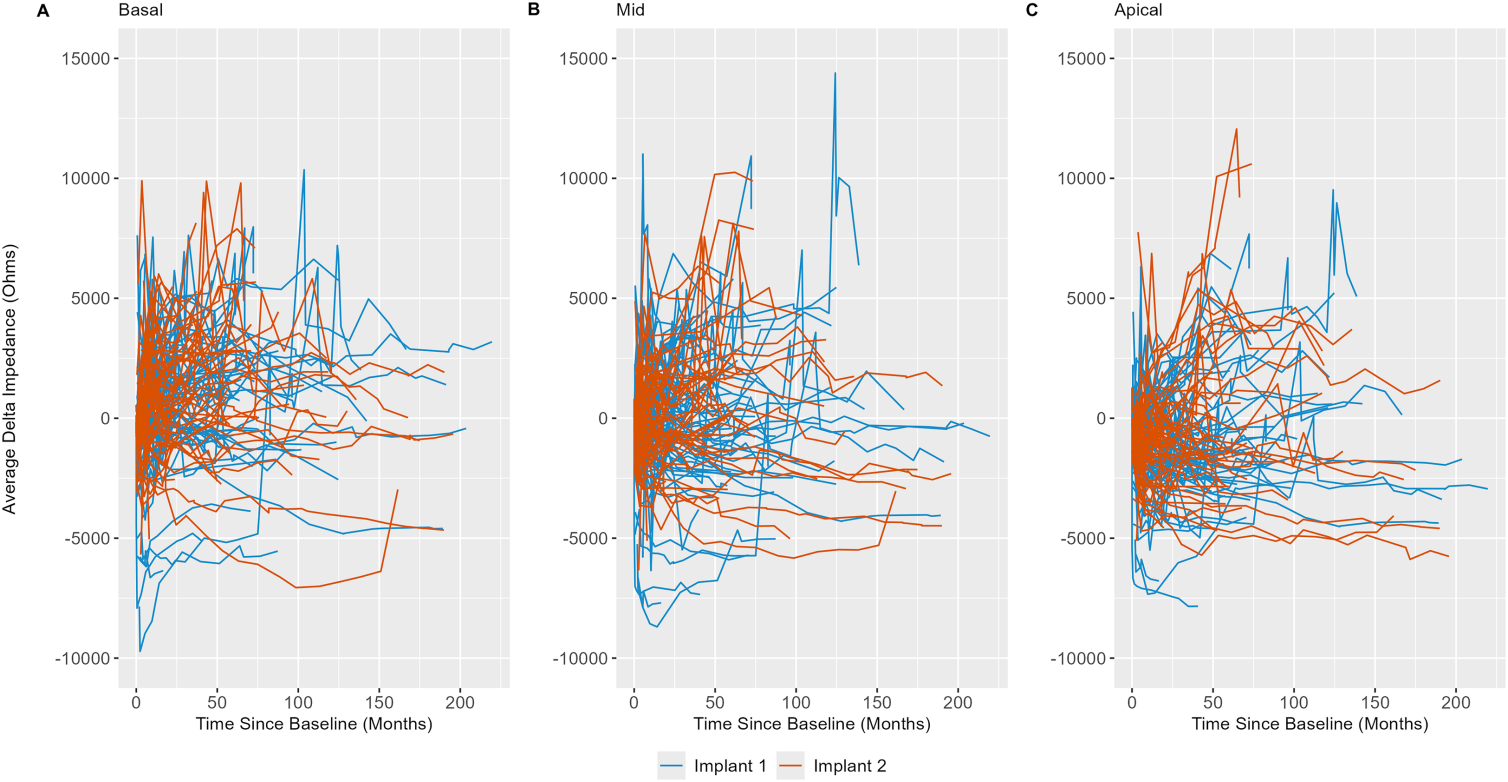
Line plots of basal (electrodes 3–6; **(A)**, middle (electrodes 10–13; **(B)**, and apical (electrodes 19–22; **(C)** electrode impedances (in Ohms) versus time relative to baseline visit (in months) by implant sequence, demonstrating within- and between-subject variability. Each line represents an individual subject in the cohort.

Delta impedance relative to the baseline visit for basal (electrodes 3–6), middle (electrodes 10–13), and apical (electrodes 19–22) subsets was plotted against time since baseline visit by implant sequence across the entire dataset (**Figure 4**), with model results shown in **Table 3**. Significant effects included implant sequence (*p* < 0.0001), time since baseline visit (*p* < 0.0001), and electrode grouping (*p* < 0.0001). Estimated marginal means (**Table 4**) indicated a statistically significant difference in delta impedance among all three electrode subsets: basal/middle (*p* < 0.0001), basal/apical (*p* < 0.0001), and middle/apical (*p* < 0.0001). Within each electrode subset, statistically significant differences in delta impedance by implant sequence were observed in the basal (*p* = 0.0136) and apical (*p* = 0.0067) groups, whereas no significant difference was seen in the middle (*p* = 0.6284) subset (**Table 5**).

**Table 3:** Linear mixed model results, where group represents electrode grouping (basal, middle, apical), Sequence represents implant sequence (first implant, second implant), Age represents age grouping as a binary variable (< 18 years, > 18 years), and time is time since baseline visit in months.

Variable	Estimate	SE	*df*	*t*-value	*p*-value
Intercept	− 520.59	208.61	4,479	− 2.50	0.0126
Group (mid)	− 108.96	129.85	4,479	− 0.84	0.4014
Group (apical)	− 906.97	129.99	4,479	− 6.98	< 0.0001
Sequence (second)	820.28	140.76	4,479	5.83	< 0.0001
Age (< 18)	− 497.37	232.11	4,479	− 2.14	0.0322
Log (time)	414.47	64.16	4,479	6.46	< 0.0001
Group (mid) * sequence	− 205.02	137.41	4,479	− 1.49	0.1358
Group (apical) * sequence	25.82	137.55	4,479	0.19	0.8511
Sequence * log (time)	− 160.36	37.19	4,479	− 4.31	< 0.0001
Group (mid) * log (time)	− 125.01	35.93	4,479	− 3.48	0.0005
Group (apical) * log (time)	− 174.57	35.93	4,479	− 4.86	< 0.0001

SE, standard error; *df*, degrees of freedom.

**Table 4:** Comparison of estimated marginal means of delta impedance by electrode subset based on the linear mixed model, including basal (electrodes 3–6), middle (electrodes 10–13), and apical (electrodes 19–22) subsets.

Comparison	Estimate	SE	*df*	*T*-ratio	*p*-value
Basal–middle	652	76.5	4,479	8.528	< 0.0001
Basal–apical	1,509	76.5	4,479	19.721	< 0.0001
Middle–apical	857	76.5	4,479	11.201	< 0.0001

SE, standard error; *df*, degrees of freedom.

**Table 5:** Comparison of estimated marginal means of delta impedance by electrode subset based on the linear mixed model, including basal (electrodes 3–6), middle (electrodes 10–13), and apical (electrodes 19–22) subsets.

Comparison	Estimate	SE	*df*	*T*-ratio	*p*-value
First–second implant | basal group	− 255	103	4,479	− 2.469	0.0136
First–second implant | middle group	− 50	103	4,479	− 0.484	0.6284
First–second implant | apical group	− 281	103	4,479	− 2.714	0.0067

SE, standard error; *df*, degrees of freedom.

**Figure 5:**
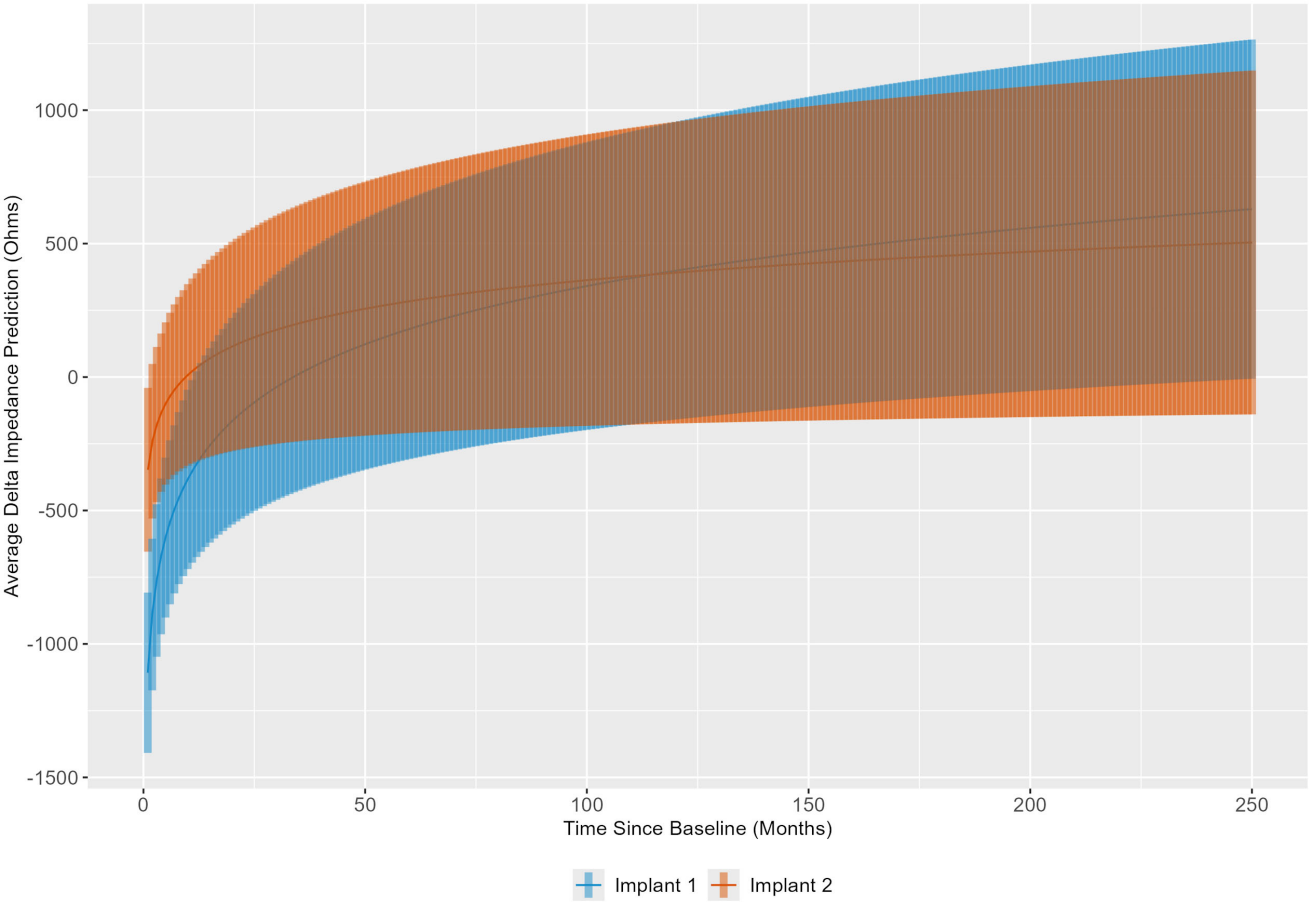
Linear mixed model results were used to illustrate the trend in delta impedance (in Ohms) over time relative to baseline visit (in months) by implant sequence.

Using the results of the linear mixed model, delta impedance for the first and second implants was computed and plotted based on the model-estimated slope for time (**Figure 5**). The results indicate that the slopes of delta impedance over time differ significantly between the first implant to the second implant (*p* < 0.0001). Corresponding confidence intervals show that the slope is steeper for the first implant, indicating a slower rate of increase in delta impedance for the second implant.

**Figure 6:**
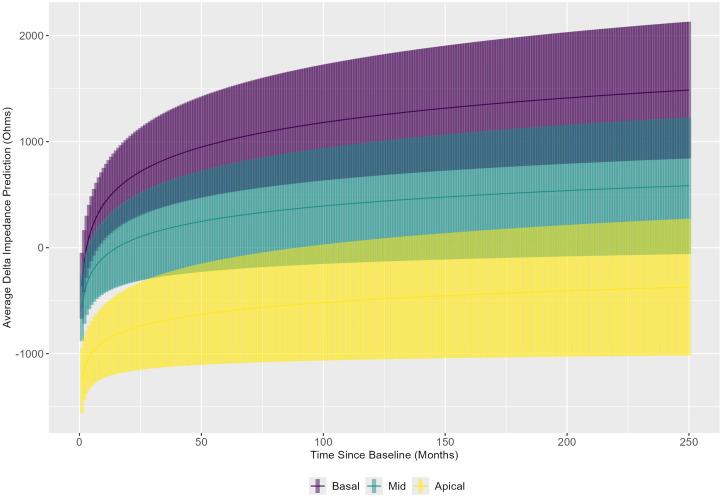
Linear mixed model results were used to show the trend in delta impedance (in Ohms) versus time relative to baseline visit (in months) by electrode subset: basal (electrodes 3–6), middle (electrodes 10–13), and apical (electrodes 19–22).

The linear mixed model results were also used to evaluate the slope over time of delta impedance for the basal, middle, and apical electrode groupings, averaged across implant sequence and age category (**Figure 6**). These results indicate that the slopes of delta impedance over time differ significantly between the basal and middle electrode groups (*p* = 0.0015). Corresponding confidence intervals show that the slope of delta impedance increases at a lower rate over time for the middle group compared to the basal group. The comparison of slopes between the basal and apical electrode groups was also significant (*p* < 0.0001), with confidence intervals indicating a slower rate of increase in delta impedance over time for the apical group relative to the basal group. The comparison between the middle and apical groups showed no significant differences (*p* = 0.3517). In summary, the slope of the delta impedance is steepest for basal electrode sites compared with middle and apical sites.

## Discussion

Here, we analyzed the spatial and temporal dynamics of electrode impedance changes in cochleae receiving sequential bilateral cochlear implants to gain insights into the inflammatory and fibrotic processes following electrode array placement. Regarding absolute impedance outcomes, these data provide a preliminary view of the dataset prior to statistical modeling. Visualization across the entire study period indicated both within- and between-subject variability in these measurements. Subsequent preliminary analyses were limited to the first 12 months of the dataset to maximize any observable effect, based on the hypothesis that reactive fibrosis or neo-ossification would likely develop within a year after implantation. Two models were used to investigate this relationship: one that averaged electrode impedance across all 22 electrodes in a standard human electrode array, and another that averaged impedance across all 22 electrodes except for the five most basal electrodes. This latter model was included *a priori* based on observations that the most basal electrodes were more likely to be deactivated by audiologists during subsequent cochlear implant interrogation visits than middle or apical electrodes. The reasons for electrode deactivation were varied and extended beyond simple dysfunction, including phenomena such as unpleasant auditory perceptions, facial nerve stimulation, absence of auditory perception despite electrode integrity, and surgical placement of electrodes outside the cochlea. The literature on electrode deactivation is mixed and largely suggests that such actions remain subjective, lacking extensive objective criteria—an observation consistent with assumptions drawn from the medical records of this patient cohort (48–50). It was therefore hypothesized that removing the five basal electrodes might accentuate any relationship in electrode impedance measures between implants. Results from these analyses demonstrated statistically significant differences in the 12-month average absolute electrode impedance between the first implantation compared to the second implantation in both models. These results provided preliminary support for the hypothesis that the second implantation exhibits greater electrode impedances. The linear mixed model further extended this finding, supporting a temporal and spatial evolution of the tissue response that begins at the base and progresses apically over time; sequential implantation appears to accelerate this response in the second implanted ear.

When comparing the estimated marginal slopes of the trends in delta impedance over time by implant sequence, the results were statistically significant. The corresponding confidence intervals indicate that the slope of delta impedance is steeper for the first implant, suggesting a slower rate of increase in delta impedance for the second implant, despite higher initial delta impedances for the second implant based on estimated marginal means in each electrode grouping. Therefore, while the second implantation demonstrates higher initial delta impedance than the first implantation, in congruence with our hypothesis, it subsequently increases at a slower rate. This finding is consistent with a role for immunological memory in accelerating the initial tissue response in the second implanted ear (higher early impedances), with less impact on the overall magnitude of the response, such that, in the long term, impedance measures are more comparable across ears. Immunological memory is based on the principle of stockpiling cells that have previously encountered damage signals or pathogens, such that they may respond to such an insult more robustly, and specifically, more quickly (51–53). It would therefore be reasonable to consider a scenario in which the second implanted cochlea experiences a burst of immunological activity that results in a quicker tissue response captured at the baseline visit, followed by a more gradual increase in delta impedance compared to the first implanted, immunologically naïve cochlea.

While not directly associated with questions related to immunological memory, another interesting trend with electrode grouping averaged over the implant sequence was discovered. In summary, it appears that the highest delta impedances are found in the basal electrode group and that the basal electrode group shows quicker increases over time in electrode impedance compared to both the middle and apical groups. Notably, the majority of human cochlear implantations occur through a round window approach, due to evidence of less severe cochlear trauma compared to a conventional cochleostomy approach (54). In both approaches, insertion-related trauma is likely greatest at the basal end of the electrode array, as this is closest to the site of entrance; this rationale may explain these basal findings in congruence with a “mechanical” hypothesis of trauma and increased electrode impedance (7, 47, 55). However, the extent to which these increases in electrode impedance may be simultaneously impacted by potential immunological foreign body response reactions also warrants consideration as a potential etiology, in congruence with an “immunological” hypothesis of increased electrode impedance *in response* to such mechanical trauma. These theories are further modulated by the potential contribution of anatomical narrowing of the scala tympani toward the cochlear apex, leading to baseline elevated apical impedances (56, 57). Further delineation of the exact contributions from these intersecting factors necessitates additional investigation that includes estimates of scalar dimensions or insertion depth when far-field measurements are evaluated, or ideally, focuses on near-field impedance measurements.

A major strength of this study was the comparison of electrode impedance between ears of the same subject, which helped control for individual differences and reduce confounding factors. This design facilitated attributing any differences to the cochlear implant sequence rather than underlying disease processes. Each subject had both ears operated on by the same surgeon, except for two who initiated care outside our center, further minimizing variability due to surgical technique differences within subjects. From a demographic standpoint, the patient cohort was well-balanced after application of the inclusion and exclusion criteria, with a near-equal distribution by sex and age group. The dataset was expansive, spanning 18 years, and the time between implantations demonstrated appropriate variability. Limitations of this study include its retrospective design, which precluded exact control of variables and patient population; the single-institution cohort; and the strict inclusion and exclusion criteria, which may reduce the generalizability of these results. While deriving the cohort from a single institution helped maintain consistency in surgical technique, perioperative care, and individual practices, some patients received their implantations at outside healthcare systems. Usage data were often sparse or entirely subjective, based on patient history at office visits, meaning this criterion may not have been applied equally to all participants; the minimal number of impedance records removed from the final cohort in this manner reflects the care taken to ensure that this criterion was as objective as possible.

Several factors not considered in this statistical analysis may be pertinent to the broader discussion regarding immunological memory and electrode impedance measurements in the context of cochlear implantation. In this patient population, the time between the first and second implants ranged widely, from 0.1 to 6.2 years. While immune memory cells are often referred to as “long-lived”, they are not immortal, and subsets of these cells decrease in number over time following the initial insult. This underlies the rationale for booster vaccinations for various infectious diseases throughout one’s lifetime (58, 59). Further consideration of this variable may be warranted in future investigations to more directly assess whether increases in impedance measurements over time from the first implantation are influenced by a potential decline in primed immunological memory cells. Another area for exploration includes characterizing potential differences between electrode array types used in these cochlear implants that employ either a lateral wall (straight) or perimodiolar array, which differ in rates of insertion trauma, electrode position within the cochlea, and sound quality (54, 60). This study included three straight (CI422, CI522, CI624) and four perimodiolar (CI24RE (CA), CI512, CI532, CI632) electrode arrays. Each participant had matched electrode array types in both ears, ensuring that these differences would not affect within-subject comparisons. However, differences between straight and perimodiolar electrode arrays may influence between-subject comparisons of electrode impedance and warrant further investigation. Similarly, the use of intraoperative dexamethasone (and more recently, dexamethasone-eluting arrays) has become commonplace in cochlear implantation to preserve residual hearing, primarily through attenuation of established fibrotic and osseous foreign body responses (61–64). Low-dose dexamethasone is also frequently administered early in the perioperative period to prevent postoperative nausea and vomiting (65). Given the time span of this dataset and the absence of complete records regarding corticosteroid administration in some cases, it is possible that patients received differential inflammatory prophylaxis due to evolving guidelines. Standardization of intralesional corticosteroids, and possibly perioperative systemic corticosteroids, may help control for potential effects on electrode impedance. Importantly, the use of these anti-inflammatory measures has increased over time in our clinics’ practice, making it unlikely that they account for the accelerated impedance changes seen in the second ear. Although not directly explored in this study, the presence of prior nonotologic implantations, such as joint replacements or implanted pacemakers, may also influence the robustness of intracochlear responses as described. Similar materials within different implants or the act of foreign body implantation itself may predispose patients to the formation of immunological memory cells that could interact across regions and tissue types; however, these possibilities require further elucidation through prospective histological and cell-based studies.

In conclusion, this study investigated the possibility that the first cochlear implant primes the body to respond more robustly to the second cochlear implant, in congruence with the principles of immunological memory, using longitudinal measures of electrode impedance as an accessible outcome variable. The tissue response adjacent to the electrode array track involves recruitment and activation of multiple immune cells, including mononuclear phagocytes and lymphocytes (12, 66, 67). Coupled with such molecular and histological observations, our results suggest that cochlear immune responses to cochlear implantation trauma and materials merit further investigation in more direct and controlled manners. Additional investigation into the impacts of timing between implants, electrode array type, cochlear surgical access techniques, and perioperative corticosteroid usage may extend our understanding of these relationships. Separation of electrode impedance into its complex subsets may also elucidate additional information regarding electrophysiological and tissue-specific characteristics. Further prospective investigation using animal models will enable more rigorous exploration of the effects of cochlear immunity on ears undergoing sequential cochlear implantation, with the added benefits of controlled ear randomization for first implantation; control over electrode activation status and the amount of electrical activation across ears and subjects; earlier and more frequent measurements across the time period immediately postimplantation; and histopathological collection and analysis. Ultimately, research in this area has the potential to inform clinical guidelines pertaining to cochlear implant sequencing and inflammatory prophylaxis in the setting of sequential cochlear implantation.

## Data Availability

The data analyzed in this study is subject to the following licenses/restrictions: University of Iowa Health Care Cochlear Implant Center Custom Sound Patient Database. Requests to access these datasets should be directed to MH, marlan-hansen@uiowa.edu.
